# qPCR Assays for the Detection and Quantification of Multiple Paralytic Shellfish Toxin-Producing Species of *Alexandrium*

**DOI:** 10.3389/fmicb.2018.03153

**Published:** 2018-12-18

**Authors:** Rendy Ruvindy, Christopher J. Bolch, Lincoln MacKenzie, Kirsty F. Smith, Shauna A. Murray

**Affiliations:** ^1^Climate Change Cluster, University of Technology Sydney, Sydney, NSW, Australia; ^2^Institute for Marine and Antarctic Studies, University of Tasmania, Launceston, TAS, Australia; ^3^Cawthron Institute, Nelson, New Zealand

**Keywords:** paralytic shellfish toxin, *Alexandrium*, qPCR, ribosomal DNA, Dinoflagellate cysts

## Abstract

Paralytic shellfish toxin producing dinoflagellates have negatively impacted the shellfish aquaculture industry worldwide, including in Australia and New Zealand. Morphologically identical cryptic species of dinoflagellates that may differ in toxicity, in particular, species of the former *Alexandrium tamarense* species complex, co-occur in Australia, as they do in multiple regions in Asia and Europe. To understand the dynamics and the ecological drivers of the growth of each species in the field, accurate quantification at the species level is crucial. We have developed the first quantitative polymerase chain reaction (qPCR) primers for *A. australiense*, and new primers targeting *A. ostenfeldii, A. catenella*, and *A. pacificum*. We showed that our new primers for *A. pacificum* are more specific than previously published primer pairs. These assays can be used to quantify planktonic cells and cysts in the water column and in sediment samples with limits of detection of 2 cells/L for the *A. catenella* and *A. australiense* assays, 2 cells/L and 1 cyst/mg sediment for the *A. pacificum* assay, and 1 cells/L for the *A. ostenfeldii* assay, and efficiencies of >90%. We utilized these assays to discriminate and quantify co-occurring *A. catenella, A. pacificum*, and *A. australiense* in samples from the east coast of Tasmania, Australia.

## Introduction

Of all paralytic shellfish toxin (PSTs) producing dinoflagellates, species of the genus *Alexandrium* are the most widely distributed around the globe, with planktonic cells found from sub-Arctic regions to the tropics (Anderson, 1997; Usup et al., 2002; McCauley et al., 2009; Brown et al., 2010; Ho et al., 2012; Gao et al., 2015a; Salgado et al., 2015). In Australia, past surveys of *Alexandrium* species indicate the presence of *Alexandrium pacificum* and *Alexandrium australiense* along the coast of the states of New South Wales (NSW) and Victoria, down to Port Lincoln in South Australia, including the east coast of Tasmania (Hallegraeff et al., 1991; Bolch and de Salas, 2007; Farrell et al., 2013; Bolch et al., 2014). Planktonic cells of *Alexandrium catenella* [previously known as *Alexandrium fundyense*, Group 1 genotype (Prud’homme and van Reine, 2017)] on the other hand, have only been detected in Tasmania, along the east coast down to the western bays of the Tasman peninsula (Bolch et al., 2014; Hallegraeff et al., 2018). *Alexandrium ostenfeldii* has been identified across estuaries in NSW (Farrell et al., 2013). In New Zealand, *A. australiense* and *A. catenella* have not been observed to date. However, in the Bay of Plenty in the North Island of New Zealand, the two PST producing species *A. pacificum* and *A. minutum* bloom regularly, especially after summer storms (Rhodes and Mackenzie, 2001; MacKenzie, 2014).

As well as living as planktonic cells, *Alexandrium* spp. undergo sexual reproduction, which results in the formation of resting cyst layers on coastal sediments (Anderson, 1998). These cysts can germinate at the right environmental conditions and become the precursor for a bloom (Bravo et al., 2010; Moore et al., 2015). The cysts of *A. ostenfeldii* and *A. pacificum* have also been detected in several sampling locations in New Zealand, and Tasmania and New South Wales states in Australia (Bolch and Hallegraeff, 1990; Mackenzie et al., 1996; Bolch, 1997; MacKenzie, 2014).

PSTs produced by *Alexandrium* species cause the human illness Paralytic Shellfish Poisoning (PSP), posing a serious public health and economic threat to the local communities worldwide (Hallegraeff, 1993). It also affects the shellfish aquaculture industry in New Zealand and Australia, which is a significant contributor to the local economy, valued at approximately USD 188 million in New Zealand and USD 100.9 million in Australia (Aquaculture New Zealand, 2012; Mobsby and Koduah, 2017). Blooms of PST-producing species have been recorded in shellfish intensive areas in Australia and New Zealand (Ajani et al., 2001, 2011; MacKenzie et al., 2004; Harwood et al., 2013), resulting in product recall and farm closures. In New Zealand, blooms of *A. pacificum* started to impact the important aquaculture region in Queen Charlotte Sound on 2011, with the numbers reaching 1.3 × 10^5^ cells/L and toxicity of 17 mg STX equivalents/kg in the mussel species *Perna canaliculus*, within a month after the first cell was observed (MacKenzie, 2014). In December 2012, a poisoning incident in the Bay of Plenty was caused by an *A. minutum* bloom (MacKenzie, 2014). Currently, the aquaculture industry is under development in this region, and the inherent risk of *Alexandrium* blooms is an important consideration. An improved understanding of the ecology of *A. pacificum* and *A. minutum*, will aid in assessing the risk of PST blooms in this region.

PST-producing blooms of *A. pacificum* and *A. catenella* have been recorded in Australia and New Zealand (Ajani et al., 2001, 2011; MacKenzie, 2014; Hallegraeff et al., 2018), and the species *A. australiense* and *A. ostenfeldii* also occur (Murray et al., 2012; Ajani et al., 2013; Farrell et al., 2013). Little is known regarding the dynamics and environmental drivers underlying blooms of each species in this region. In particular cases, more than one species can co-occur during a bloom (Hallegraeff et al., 2018). Co-occurrences of cryptic *Alexandrium* species during blooms have also been reported in the United Kingdom (Toebe et al., 2013; Eckford-Soper et al., 2016), Ireland (Touzet et al., 2010), the Mediterranean (Penna et al., 2008) and Korea (Shin et al., 2017). Environmental factors such as seasonality, temperature, rainfall, parasitism and other factors influencing the growth, development and decline of blooms of *Alexandrium* species is species-specific, and therefore, accurate quantification at the species level is vital (MacKenzie et al., 2004; Orr et al., 2011; Murray et al., 2012; Hackett et al., 2013).

In general, the few previous studies of *Alexandrium* in Australia have used light microscopy to identify species (Hallegraeff et al., 1991; Bolch and de Salas, 2007; Farrell et al., 2013). One of the crucial limitations of this method is its inability to discern species with morphological features that are not distinguishable, or overlap, which is the case for *A. pacificum, A. catenella*, and *A. australiense*. While these species are not distinguishable based on their morphology, they have been identified as part of the former *A. tamarense* species complex and can be distinguished by clear genetic differences (Scholin et al., 1994; John, 2004; Lilly et al., 2007; John et al., 2014). For cyst identification, the use of optical microscopy may also not be the most reliable method, as the cyst morphology can be very similar for species within the genus *Alexandrium* (Kamikawa et al., 2005) and species confirmation is required through germination. Therefore, a method that is able to detect and identify the species of *Alexandrium* cysts will be useful.

The development of molecular genetic methods to detect and enumerate harmful algal bloom forming species, such as microarrays, next generation sequencing, and quantitative Polymerase Chain Reaction (qPCR) has been rapid (Lilly et al., 2007; Ebenezer et al., 2012; John et al., 2014; Medlin and Orozco, 2017). Apart from their sensitivity, molecular methods allow for high throughput sample analysis. Platform such as qPCR have grown in popularity due to their sensitivity and specificity (Penna and Galluzzi, 2013), as well as their speed and lower cost in comparison to light microscopy-based identification (Dias et al., 2015). Species-specific qPCR assays have been developed to identify cysts and motile cells of *A. catenella, A. pacificum*, and *A. ostenfeldii* utilizing ribosomal gene regions as targets (Hosoi-Tanabe and Sako, 2005; Erdner et al., 2010; Vandersea et al., 2017). However, no assay has yet been developed for *A. australiense*, and the specificity of previously published assays has not been thoroughly tested in the laboratory. The only published assay for detecting *A. ostenfeldii* was not ideal, as it did not show a strong correlation with cell counts through other methods (*R*^2^ = 0.44, Vandersea et al., 2017).

An assay based on a functional gene target involved in PST biosynthesis (*sxtA4*) has been developed (Murray et al., 2011; Gao et al., 2015b; Penna et al., 2015). This method has shown high levels of efficiency and specificity to PST producing dinoflagellates, and has also been trialed in shellfish meat as an indicator of meat toxicity (Farrell et al., 2016). The qPCR assay based on *sxtA* outperformed qPCR methods targeting rDNA in several ways, such as the consistency of results among sites (Murray et al., 2011; Gao et al., 2015b; Penna et al., 2015), and is therefore useful as a monitoring tool. However, it does not allow for the identification of individual species, which is required in order to investigate the ecological parameters related to bloom formation of individual species.

In this study, we developed and analyzed the specificity and the efficiency of qPCR species-specific assays targeted at the species known to be present in Australian and New Zealand waters, *A. catenella, A. pacificum, A. ostenfeldii*, and for the first time *A. australiense*. For the *A. catenella* and *A. pacificum* assays, the specificity and sensitivity were compared to previously published assays. The performance of the qPCR species-specific assays in relation to light microscopy cell and cyst counts was also assessed, to explore the possibility of using this assay for the detection of sediment cysts.

## Materials and Methods

### *Alexandrium* Culture Conditions

*Alexandrium* cultures were grown at 35 PSU (Practical Salinity Unit) salinity, light was provided on a 12 h/1 2h dark/light cycle at a photon irradiance of 100 μmol photons m^2^s^-1^ (Table 1). Strains used in this study were provided by the Cawthron Institute Culture Collection of Micro-algae, Australian National Algae Culture Collection, and University of Tasmania (Isolated by Chris Bolch and Miguel de Salas).

**Table 1:** Cultures used for this study.

Species Name	Strain number	Origin	Isolation date	Isolator	Growth temperature	Growth media
*A. pacificum*	CAWD44	Tauranga, NZ	November 97	L. MacKenzie/J. Adamson	18	GSe
	CS300	Samchonpo, Korea	November 90	C. Bolch	18	GSe
*A. australiense*	ATCJ33			Miguel de Salas	18	GSe
	AT-YC-H			C. Bolch	18	GSe
*A. catenella*	TRIA-E	Triabunna, Tasmania, AU		C. Bolch	18	GSe
	AT-STH-M	St Helen, Tasmania, AU		C. Bolch	18	GSe
*A. ostenfeldii*	CAWD136	Big Glory Bay, NZ	January 04	L. Mackenzie	18	GP
	CAWD135	Big Glory Bay, NZ	January 04	L. MacKenzie	18	GP
*A. minutum*	CAWD12	Anakoha Bay, NZ	January 94	L. MacKenzie	18	GSe
	CS324/12	Port River, Adelaide, AUS	November 88	S. Blackburn/J. Cannon	18	GSe
	AMNC04	Newcastle, NSW	August 97	C. Bolch	18	GP

### Development of qPCR Assays

Ribosomal sequences from the large subunit 28S of four *Alexandrium* species were examined for regions of similarity and difference. Comprehensive alignment were assigned for all examined species from all available strains. Regions of similarity were defined by eye. Informative sequence differences were identified based on conserved regions from each species, and primers were designed at these locations. Five sets of primers and probes intended to be specific for each of the five *Alexandrium* target groups were designed using Geneious R11 software. Probes were labeled with 6 FAM (6-carboxyfluorescein) to the 5′-end of the sequences, and BHQ1 (Black Hole Quencher) was attached to the 3′-end as a quencher (Integrated DNA Technologies, IA, United States).

Cross-reactivity was tested using qPCR carried out on a StepOne Plus Real-Time PCR System (Thermo Fisher Scientific, MA, United States) platform with the following cycles: 95°C for 10 s and 35 replicates of 95°C for 15 s and 60°C for 30 s. Each 20 μL reaction contained 10 μL of Itaq Universal Probe mix (Bio-Rad, CA, United States), 0.5 μM of each primers (Table 2), 0.05 μM fluorescence probe, 1 μL template DNA (correspond to 500–1000 cells) 1 μL BSA, and 6 μL PCR-grade water.

**Table 2:** Sequence of primers and probes specific to *A. catenella, A. pacificum, A. australiense*, and *A. ostenfeldii*.

Target species	Reference	Primers	
		Name	Sequence
*A. catenella*	This publication	ACT-US-408-F	5′-ACT TGA TTT GCT TGG TGG GAG-3′
		ACT-US-645-R	5′-AAG TCC AAG GAA GGA AGC ATC C-3′
	This publication	US-412-F	5′-TGA TTT GCT TGG TGG GAG TG-3′
		US-642-R	5′-CAA GGA AGG AAG CAT CCC C-3′
		US-443-P	FAM-CTTGACAAGAGCTTTGGGCTGTG-BHQ1
	Erdner et al., 2010	AlexLSUf2	5′-GGC ATT GGA ATG CAA AGT GGG TGG-3′
		Alexgp1RevAF1	5′-GC AAG TGC AAC ACT CCC ACC AAG CAA-3′
*A. pacificum*	Hosoi-Tanabe and Sako, 2005	catF	5′-CCT CAG TGA GAT TGT AGT GC-3′
		catR	5′-GTG CAA AGG TAA TCA AAT GTC C-3′
	This publication	ACTA-416-F	5′-TCC TCA GTG AGA TTG TAG TG-3′
		ACTA-605-R	5′-GAC AAG GAC ACA AAC AAA TAC-3′
		ACTA456-P	FAM-TTTGGCTGCAAGTGCAATAATTCTT-BHQ1
*A. australiense*	This publication	AusTv2-F	5′-CGG TGG GTG CAA TGA TTC-3′
		AusTv2-R	5′-GCA GGA AAA TTA CCA TTC AAG T-3′
		AusTv2-P	CACAGGTAATCAAATGTCCACATAGAAACTG
*A. ostenfeldii*	This publication	AO352-F	5′-AAACAGAATTGATCTACTTGGTG-3′
		AO493-R	5′-ATTCCAATGCCCACAGG-3′
		AO377-P	FAM-ATTGTTGCGTCCACTTGTGGG-BHQ1

Primer specificity tests were carried out by running qPCR reactions against DNA templates from *A. catenella, A. pacificum, A. australiense, A. minutum*, and *A. ostenfeldii* (Table 3). At the exponential phase, a total of 60,000–75,000 cells were harvested in triplicate by centrifugation using Gyrozen 1580R centrifuge at 1,000 *g* for 10 min (LaboGene, Bjarkesvej, Denmark). FastDNA Spin kit for Soil (MP Biomedicals, Solon, OH, United States) was used to extract the DNA from the cultures according to manufacturer’s protocol. Samples were eluted in 80 μl of elution buffer and stored at -20°C until further analysis. The quantity and quality of the DNA was measured by Nanodrop ND-1000 (Thermo Fisher Scientific, MA, United States).

**Table 3:** Specificity of *Alexandrium* qPCR Assays.

Species	Assays	*A. pacificum*		*A. australiense*		*A. catenella*		*A. ostenfeldii*		*A. minutum*	
		CAWD44	CS300	ATCJ33	AT-YC-H	TRIA-E	AT-STH-M	CAWD135		CAWD12	CS324
*A. catenella*	ACT-US-408-F/ACT-US-645-R	–	–	–	–	+	+	–		–	–
	US-412-F/US-642-R	–	–	–	–	+	+	–		–	–
	AlexLSUf2/Alexgp1RevAF1	–	–	–	–	+	+	–		–	–
*A. pacificum*	catF/catR	+	+	+	–	–	–	–	–	–
	ACTA-416-F/ACTA-605-R	+	+	–	–	–	–	–		–	–
*A. australiense*	AusTv2-F/AusTv2-R	–	–	+	+	–	–	–		–	–
*A. ostenfeldii*	AO352-F/AO493-R	–	–	–	–	–	–	+		–	–

*+, Target sequence amplified; -, target sequence not amplified.*

The reaction efficiency of each primer pairs was determined through the slope of the log-linear portion of the cell-based standard curve from one strain from each species. Ten-fold serial dilution of the DNA extracts at a range between 2000 and 0.02 cells for *A. catenella, A. pacificum*, and *A. australiense* and between 800 and 0.008 cells for *A. ostenfeldii* were used to develop the curves. Three replicate DNA extracts from each strain were used to construct the standard curves. The qPCR was carried out as described above.

### Test of qPCR Methodology for Cyst Quantification

A van Veen grab (320 mm × 350 mm sampling area) was used to obtain sediment samples in shellfish aquaculture intensive area in Opua Bay, Marlborough Sounds, New Zealand. The grab had a lid that prevented surface sediments being lost on retrieval. Sediment within the grab sample was subsampled with three 60 mm diameter cores from which the top 1 cm was sliced off and pooled to make one composite sample. From this sample, the number of cysts per m^2^ of sediment in the surface sediments could be calculated. Samples were stored frozen at -20°C after collection as freezing does not affect the integrity of dinoflagellate cysts in sediment samples (Mertens et al., 2012).

*Alexandrium pacificum* cysts were counted using the fluorescent staining method described by Yamaguchi (Yamaguchi et al., 1995). Briefly, this involved diluting 2 mL of the homogenized sediment sample in tap water, sonicating and prefiltering through an 80 μm mesh to remove larger particulates. The filtrate was washed through a 20 μm screen and the deposit made up to 25 mL. A 5 mL subsample was fixed with 100 μL of glutaraldehyde, centrifuged and the pellet resuspended in 15 mL of methanol and stored at 4°C for a minimum of 4 h (less than 4 h produces variable staining and inaccurate counts). After centrifugation and washing with distilled water, the pellet was stained for 1 h with a 2 mg mL^-1^ solution of Primuline dye (Sigma-Aldrich 206856-5G). After further washing with distilled water and resuspension in 5 mL of water, a subsample (typically 0.25–0.5 mL) was settled in an Utermöhl chamber and examined under UV light with an inverted microscope. Using this method, the brightly stained *A. pacificum* cysts contrasted well against the background of other particulate material in the sample and were easily identified and counted.

For qPCR analysis, 50 mg subsamples of sediment were taken for total genomic DNA extractions using Power Soil^®^ DNA isolation kits (Qiagen, Valencia, CA, United States) following the manufacturer’s protocol. DNA extractions were eluted into 50 μL, quantified using a NanoPhotometer (Implen, Munich, Germany) to check for DNA quantity and quality (260/280 ratio), and stored at -20°C until further analysis.

### Cross Depth Quantification of *Alexandrium* spp

Seawater samples were collected during the peak of an *A. catenella* bloom in the shellfish farming area on the east coast of Tasmania in Spring Bay and Great Oyster Bay in August 2016 aboard the RV *Southern Cross* (Figure 2). Seawater samples of 3 L were collected at multiple depths with Niskin bottles on the same location and filtered with 8 μm polycarbonate filters in triplicates and stored at -20°C until the DNA extraction. DNA extraction was then carried out with FastDNA for Soil kit according to manufacturer’s protocol (MP Biomedical, United States) and stored at -20°C until analysis.

One liter of water was stored in Lugol’s iodine at 4°C until *Alexandrium* cell concentrations were calculated. The samples were settled and total *Alexandrium* cells were counted using inverted microscope (Leica Microsystems, Wetzlar, Germany).

### Bloom Dynamics Comparative Analysis Between Light Microscopy and qPCR

**FIGURE 1:**
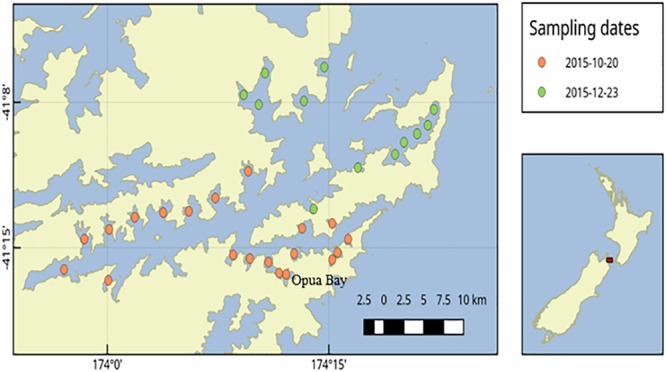
Sediment sampling stations in Queen Charlotte Sound, Marlborough Sounds, New Zealand.

During a bloom of *A. pacificum* in Opua Bay (Queen Charlotte Sound, the Marlborough Sounds, New Zealand) samples were collected for both light microscope counts and qPCR analysis (Figure 1). Discrete grab samples were collected from 6 m depth. Samples for microscope cell counts (100 mL) were preserved in Lugol’s iodine and a subsample of 10 mL was settled in an Utermöhl chamber and examined with an inverted microscope (CK41, Olympus, Wellington, New Zealand). For qPCR analyses 100 mL of seawater was filtered (Durapore membrane filters, 0.45 μm, Millipore, United States). Genomic DNA was extracted from the filter papers using Power Soil DNA extraction kits as described above. The genomic DNA samples were then analyzed using the *A. pacificum* qPCR assay as described above. To create a standard curve for the qPCR analyses DNA was extracted from replicate samples of known numbers of cells of the CAWD44 strain of *A. pacificum*. Cell concentrations of culture were estimated during exponential growth phase using Utermöhl chambers and an inverted microscope. Replicates samples consisting of 150,000 cells were filtered (Durapore membrane filters, 0.45 μm) and extracted using Power Soil DNA extraction kits (Qiagen, Hilden, Germany) as described above. These extractions were serially diluted and used to generate a standard curve of known cell number per reaction versus Ct data.

**FIGURE 2:**
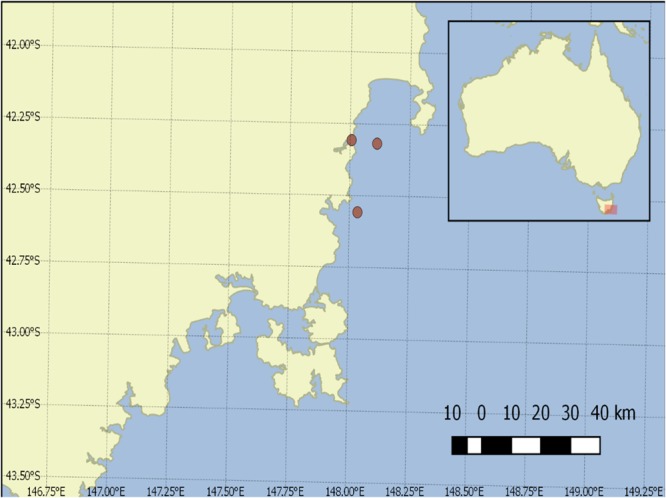
Sample collection points in Great Oyster Bay and Spring Bay, Tasmania.

## Results

### *Alexandrium* sp. Species-Specific Assays Specificity Test

In this study, we designed two primer pairs and a probe for *A. catenella*, and one primer pair and probe each for *A. catenella, A. australiense* and *A. ostenfeldii* species (Table 2). The specificity of the assays and previously published assays was tested. Except for a previously published catF/catR assay (Hosoi-Tanabe and Sako, 2005), which positively amplified the strain of *A. australiense*, all the tested assays were specific to the target species (Table 3).

Further BLAST (Basic Local Alignment Tool) nucleotide analysis of catF/catR primers resulted in 100% match of the reverse primer with *A. australiense* strain ATCJ33 (KJ879221.1) internal transcribed spacer 1, partial sequence; 5.8S ribosomal RNA gene, complete sequence; and internal transcribed spacer 2, partial sequence.

### *Alexandrium* sp. Species-Specific Assays Efficiency Assessment

**FIGURE 3:**
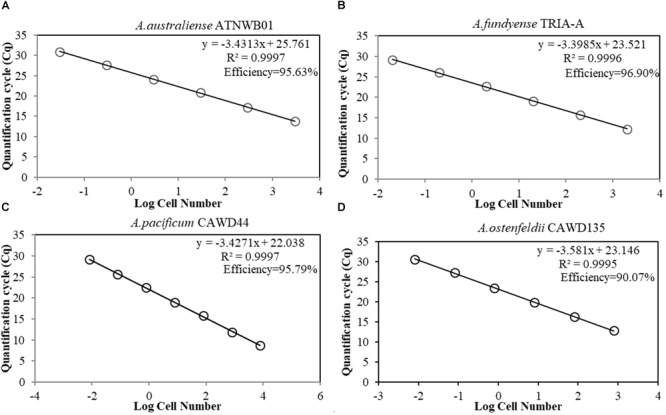
**(A–D)** Standard curves of species-specific assays for *Alexandrium* spp., presenting the quantification cycle (*y*-axis) versus the known cell number in log scale (*x*-axis). **(A)** *A. australiense* strain ATNWB01 standard curve. **(B)** *A. catenella* strain TRIA-A standard curve. **(C)** *A. pacificum* strain CAWD44 standard curve. **(D)** *A. ostenfeldii* strain CAWD135 standard curve.

The amplification efficiency of the assays was measured based on the standard curve slope, developed through serial dilution of DNA extracts from known cell numbers. The reaction efficiencies for all tested assays were higher than 90%. Coefficient of determination of all standard curves were higher than 0.99. The reaction efficiency of the assay targeting *A. ostenfeldii* was lower than assays specific for *A. catenella, A. pacificum*, and *A. australiense* (Figure 3). The limits of detection were 2 cells/L for the *A. catenella* and *A. australiense* assays, 2 cells/L and 1 cyst/mg sediment for the *A. pacificum* assay, and 1 cells/L for the *A. ostenfeldii* assay.

**FIGURE 4:**
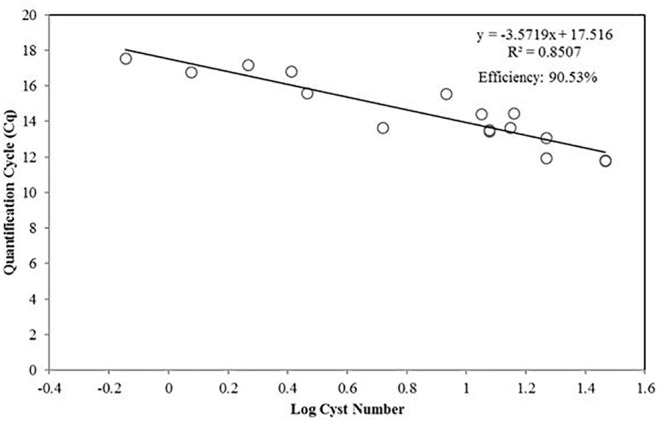
Standard curve presenting the qPCR quantification cycle value (*y*-axis) versus the diluted number of *A. pacificum* cysts in log scale.

The *A. pacificum* assay (ACTA-416-F/ACTA-605-R) was tested with DNA extracts from cysts (Figure 4). The efficiency of the reaction was lower when quantifying planktonic cells but sufficiently high (90.53%), and the coefficient of determination was also relatively high (0.8507).

### Comparison Between Light Microscopy and qPCR

**FIGURE 5:**
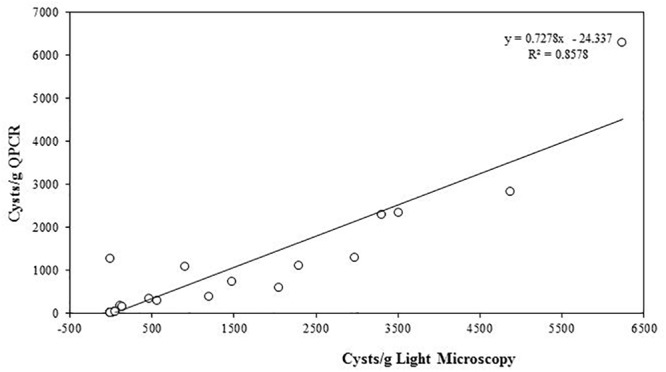
Linear regression of *A. pacificum* cyst quantification from sediments with light microscopy and qPCR.

The quantity of *A. pacificum* cysts per gram of sediment samples from the same source were quantified using both light microscopy and qPCR. The coefficient of determination (Pearson *R*^2^ = 0.8578) shows a high correlation between the value obtained from light microscopy and qPCR (Figure 5).

**FIGURE 6:**
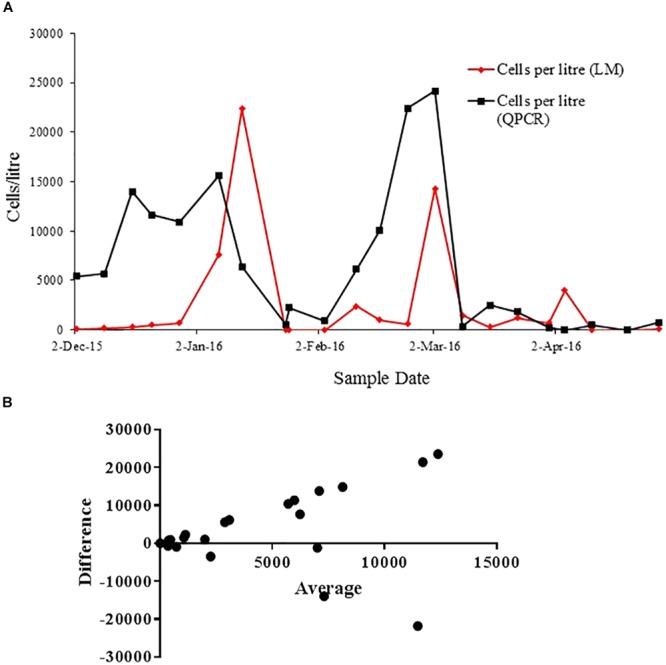
**(A)** Correlation of cells quantification with light microscopy (red line) and qPCR method (black line) throughout the *A. pacificum* bloom in Opua Bay, Marlborough Sound. **(B)** Bland-Altman measurement of agreement between cell quantification using light microscopy and species-specific qPCR assay.

The cell count using light microscopy was compared with the quantification using qPCR approach in samples from Opua Bay (Figure 6A). The cell number fluctuation observed with the light microscopy and qPCR methods are comparable, especially in low cell number (below 5000). On the date of December 2nd, 2015, about 100 cells were detected using light microscopy and 5.4 × 10^3^ cells were detected with qPCR. On March 2nd, 2016, 1.4 × 10^4^ cells were detected by light microscopy, and 2.4 × 10^4^ cells were detected by qPCR. During times of the highest cell concentration, the qPCR assay detected more cells than light microscopy. The results from the Bland-Altman analysis (Figure 6B) supported this observation, showing less agreement between two methods in the high cell number phase (above 5000 cells/L).

**FIGURE 7:**
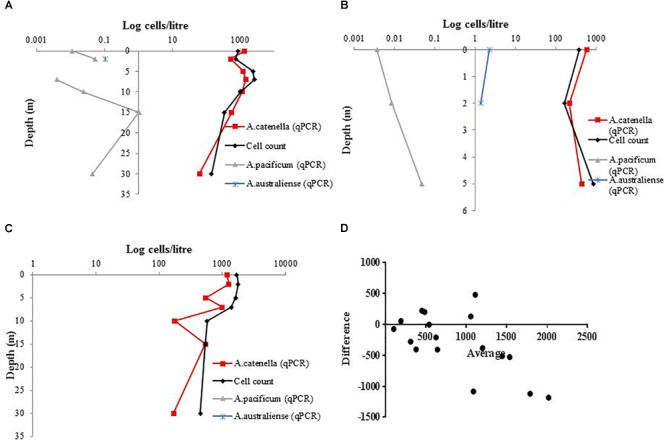
Cross depth cell quantification using species-specific qPCR assays for *A. catenella, A. pacificum*, and *A. australiense*, in comparison with total *Alexandrium* count **(A)**. Great Oyster Bay Site 1 **(B)** Great Oyster Bay Site 2. **(C)** Spring Bay Site 3 **(D)** Bland-Altman measurement of agreement between species-specific *A. catenella* assays and light microscopy.

The cross-depth cell quantification on samples from Spring Bay and Great Oyster Bay, Tasmania, using light microscopy and species-specific qPCR assays suggest that the bloom was dominated by *A. catenella* (Figures 7A–C). The quantity of cells measured by light microscopy and qPCR appeared to correlate well. Additionally, Bland-Altman analysis of combined Southern Cross samples shows that the differences between two methods are mostly around 500 cells/L, with the plots spread evenly (Figure 7D).

## Discussion

The co-occurrence of cryptic species of dinoflagellates can lead to difficulties in accurately enumerating the target species, which is necessary in order to establish the ecological factors driving the growth, proliferation and decline of harmful algal blooms. Rapid molecular methods have become a promising solution (Touzet et al., 2009, 2010; Toebe et al., 2013). Currently, the occurrence and seasonality of *A. australiense* blooms in Australia or elsewhere have never been established. *A. australiense* can be weakly toxic or non-toxic (Murray et al., 2012), as two of the four strains for which PSTs have been measured have shown very low levels of PSTs. Understanding the bloom dynamics and environmental drivers may potentially reveal the factors underlying intra-species toxicity variation. This study described the first method for determining the abundance of *A. australiense*, with sensitivity up to one cell and efficiency of 95.63% (Figure 3A). The assay was also specific to this species, when tested against DNA extracts from *A. catenella*, and *A. pacificum* and samples.

The specificity test (Table 3) indicated that our *A. pacificum* assay is more specific when compared to a previously published assay (Hosoi-Tanabe and Sako, 2005). Further BLAST analysis of the primer pair revealed an overlap between *A. australiense* long subunit sequences with the reverse primer. This shows that we could find not just morphological, but also genetic sequence overlaps in cryptic species, emphasizing the importance of cross-specificity test against species from this complex. All the assays showed high efficiency (Figure 3), and all the assays showed the limit of detection of 2 cells/L for *A. catenella, A. pacificum, A. australiense* assays, and 2 cells/L for *A. ostenfeldii* primers. The efficiency of the *A. ostenfeldii* assay is lower (90.07%), when compared to other tested assays (above 95%). However, it is still between the accepted range (90–100%) (Vandersea et al., 2017).

Cyst are usually found in sediment as dormant cells, and can persist for decades (Anderson et al., 2005). The detection and enumeration of cysts can provide information to determine the potential risk of toxic blooms in a certain area, and possibly the scale of it. Our results strongly indicated that quantification using qPCR is comparable to a widely used auto fluorescence methodology (Yamaguchi et al., 1995). One of the advantages of the qPCR method is its relative simplicity and high-throughput capacity, allowing simultaneous quantification of cysts across different samples. However, phenolic substances and humic acids commonly found in sediment samples could inhibit amplification reactions (Roose-Amsaleg et al., 2001), lowering the efficiency of the qPCR reaction. This is shown by the lower standard curve efficiency when used to quantify diploid cysts compare to haploid planktonic cells (Figures 3C, 4). The extraction protocol we used, and the PCR condition appear to be optimal, as can be shown from the above 90% efficiency (Figure 4).

The assays that we have designed for *A. catenella, A. pacificum*, and *A. australiense* were used jointly in order to characterize the species responsible for a bloom event in Tasmania, Australia (Figures 7A–C). In this case, the 2016 bloom on the east coast of Tasmania was dominated by *A. catenella*, while *A. pacificum* and *A. australiense* were also detected in the background. This pattern is consistent with the other samples analyzed in 2015 in the east coast of Tasmania (Hallegraeff et al., 2018). Quantification of an *A. pacificum* bloom over time suggest that the results of the qPCR assays were comparable to those of the microscope cell count in terms of tracking the patterns over time of the bloom development and collapse (Figure 6A). Nonetheless, we found that the qPCR methods based on rRNA genes overestimated the absolute cell abundance compared to the count using light microscopy (Figures 6A,B). The overestimation of *Alexandrium* cell abundances using species-specific qPCR assays based on ribosomal RNA genes has been previously reported (Godhe et al., 2007). Ribosomal RNA genes copy number difference across strain can be within orders of magnitude (Galluzzi et al., 2010; Savela et al., 2016). It is possible that the copy number of the strain used for the standard curve were very different to that of the environmental strains. Consequently, the difference was amplified when the cell number increased. Another Bland-Altman analysis of the Tasmanian bloom samples (Figure 7D) shows that there is lesser overestimation when local strain is used for qPCR quantification. Thus, the use of multiple local strains to develop the standard curve may be able to correct the impact of the variation in copy number of the target gene. Alternatively, when species-specific information is not the crucial need for a study, the use of an assay based on functional gene target related to PST synthesis (e.g., *sxtA4*) (Murray et al., 2011; Gao et al., 2015b; Penna et al., 2015) can be an alternative, as the copy number variation among strains appears to be far less for this gene region than that of rRNA genes (Murray et al., 2011; Savela et al., 2016).

## Conclusion

In conclusion, we describe the assay based on qPCR for distinguishing the cryptic species *A. australiense* from potential co-occurring bloom with morphologically identical species *A. pacificum* and *A. catenella*, as well as the toxic species *A. ostenfeldii*. We show that this method can be used to estimate abundance of these potentially PST-producing dinoflagellates in mixed samples. We have also shown that the methodology can be used to quantify cysts with comparable precision to current microscopy-based method.

## Author Contributions

RR, CB, LM, KS, and SM conceived the idea. RR, LM, and KS performed the field sampling and qPCR experiments. CB isolated and maintained part of the culture used in this project. RR, LM, KS, and SM analyzed the data. RR and KS wrote the manuscript sections. All the authors reviewed and approved the manuscript.

## Conflict of Interest Statement

The authors declare that the research was conducted in the absence of any commercial or financial relationships that could be construed as a potential conflict of interest.
